# Survey on prevalence and risk factors on HIV-1 among pregnant women in North-Rift, Kenya: a hospital based cross-sectional study conducted between 2005 and 2006

**DOI:** 10.1186/1472-698X-9-10

**Published:** 2009-04-30

**Authors:** Michael Kiptoo, Solomon Mpoke, Zipporah Ng'ang'a, Jones Mueke, Fredrick Okoth, Elijah Songok

**Affiliations:** 1 Centre for Virus Research, Kenya Medical Research Institute, Nairobi, Kenya; 2 Department of Zoological Sciences, Kenyatta University, Nairobi, Kenya; 3 Department of Medical Laboratory Sciences, Institute of Tropical Medicine and Infectious Disease, Jomo Kenyatta University of Agriculture and Technology, Nairobi, Kenya; 4 Centre for Biotechnology Research & Development, Kenya Medical Research Institute, Nairobi, Kenya

## Abstract

**Background:**

The HIV/AIDS epidemic in Kenya is a major public-health problem. Estimating the prevalence of HIV in pregnant women provides essential information for an effective implementation of HIV/AIDS control measures and monitoring of HIV spread within a country. The objective of this study was to determine the prevalence of HIV infection, risk factors for HIV/AIDS and immunologic (lymphocyte profile) characteristics among pregnant women attending antenatal clinics in three district hospitals in North-Rift, Kenya.

**Methods:**

Blood samples were collected from pregnant women attending antenatal clinics in three district hospitals (Kitale, Kapsabet and Nandi Hills) after informed consent and pre-test counseling. The samples were tested for HIV antibodies as per the guidelines laid down by Ministry of Health, Kenya. A structured pretested questionnaire was used to obtain demographic data. Lymphocyte subset counts were quantified by standard flow cytometry.

**Results:**

Of the 4638 pregnant women tested, 309 (6.7%) were HIV seropositive. The majority (85.1%) of the antenatal attendees did not know their HIV status prior to visiting the clinic for antenatal care. The highest proportion of HIV infected women was in the age group 21–25 years (35.5%). The 31–35 age group had the highest (8.5%) HIV prevalence, while women aged more than 35 years had the lowest (2.5%).

Women in a polygamous relationship were significantly more likely to be HIV infected as compared to those in a monogamous relationship (p = 0.000). The highest HIV prevalence (6.3%) was recorded among antenatal attendees who had attended secondary schools followed by those with primary and tertiary level of education (6% and 5% respectively). However, there was no significant relationship between HIV seropositivity and the level of education (p = 0.653 and p = 0.469 for secondary and tertiary respectively). The mean CD4 count was 466 cells/mm^3 ^(9–2000 cells/mm^3^). Those that had less than 200 cells/mm^3 ^accounted for 14% and only nine were on antiretroviral therapy.

**Conclusion:**

Seroprevalence of HIV was found to be consistent with the reports from the national HIV sentinel surveys. Enumeration of T-lymphocyte (CD4/8) should be carried out routinely in the antenatal clinics for proper timing of initiation of antiretroviral therapy among HIV infected pregnant women.

## Background

HIV/AIDS is the major health priority in Kenya. Sentinel surveillance in antenatal women has been used to project national HIV seroprevalence in Kenya since 1990 [1]. The system operates within antenatal clinics situated in urban and peri-urban or rural sites around the country, that offer care to women during pregnancy. Each year, pregnant women in each site are anonymously tested for HIV.

The estimate for prevalence in Kenya at the end of 2003 was 6.7%, following refinement of the models used for calculations to include data from household surveys rather than just sentinel surveillance data [2]. In the preliminary results of the 2007 Kenya AIDS Indicator Survey (KIAS), the overall prevalence rate for the country was estimated to be 7.8% for Kenyans ages 15–49 and 7.4% for ages 15–64. It is estimated that 2.2 million Kenyans are now living with HIV infection, but few know whether they are infected or show outward symptoms of the disease.

This study was part of a major protocol determining the prevalence of HIV nevirapine resistant genotypes and their effects on mother-to-child transmission among antenatal clinic attendees in three district hospitals in North Rift Valley Province of Kenya. The North Rift region was chosen because it lacked documented risk factors for HIV transmission. Kitale hospital is an existing sentinel site under the National HIV/AIDS Control Programme, while Nandi hills and Kapsabet hospitals were included as new sites for the study. The latter hospitals were included since no information on the HIV prevalence was available. Generating local epidemiological and socio-demographic data of HIV infection is of critical importance in planning of interventions in the area.

## Methods

### Setting

The study was carried out from April 2005 to September 2006 at Kitale, Kapsabet and Nandi hills district hospitals.

### Participants

All pregnant women attending the antenatal clinic for the first time during the current pregnancy were included. Voluntary counseling and testing (VCT) for HIV among pregnant women has been integrated into maternal child health (MCH) facilities in all the public hospitals as part of prevention of mother to child transmission (PMTCT) of HIV. All the pregnant women attending the antenatal clinics were sensitized with basic knowledge on HIV/AIDS. The importance of knowing their HIV status and the availability of measures to reduce the risk of mother to child transmission were explained in greater detail. The objectives of the study were explained and informed consent was obtained by signature or finger print from ANC attendees. Enrolment and counseling were done in all the three hospitals.

This study was approved by the Kenya Medical Research Institute Scientific Steering Committee and Ethical Review Board (Ref. SSC No. 822). The study was conducted according to the national and international regulations governing the use of human subjects in biomedical research.

### Data collection tool

A pre-tested standard structured questionnaire was used for interview. The information sought included basic demographic data.

### Specimen

Routine investigations in the antenatal clinic necessitate blood withdrawal for HIV testing. A small amount of this blood was used also for enumeration of T-lymphocytes, which is not done routinely. Five milliliters venous blood sample was collected in a sterile vacutainer tube containing EDTA as anticoagulant from all pregnant women.

### Serology

HIV antibodies were tested by rapid tests as per the guidelines laid down by the Ministry of Health, Kenya. Rapid parallel testing was carried out using Determine™ HIV-1/2 (Abbott Diagnostic Division) and Uni-Gold™ HIV (Trinity Biotech) test kits. In case of discrepancy, Bioline HIV 1/2 3.0 (Standard Diagnostics) was used as a tiebreaker.

### Lymphocyte subset counting

Lymphocyte subset counts were performed by standard flow cytometry. The details of the procedure were performed in accordance with the manufacturer's instructions (Tritest; Becton-Dickinson, Franklin Lakes, NJ). Briefly, 50 μl of whole blood with EDTA were incubated with three-color fluorochrome-labeled monoclonal antibodies. After lysis and incubation, flow cytometric analysis was performed on a FACSCalibur cytometer using an automatic acquisition and analysis program (Multiset; Becton-Dickinson). The absolute CD4 T cell counts were recorded and used in this study.

### Statistical analysis

Data were entered using Microsoft Access (Microsoft Corporation, Redmond, Washington) and statistical analyses performed using EPI INFO 3.2.2 statistical package. We present odds ratios (OR), and 95% confidence interval (CI) for factors associated with HIV infection.

## Results

Data were collected and analyzed from a total of 4,638 pregnant women attending antenatal care in Kitale, Kapsabet and Nandi Hills district hospitals. Out of these 26.8% were from Kitale, 31.2% from Kapsabet and 41.9% from South Nandi Hills. Table 1 presents the socio-demographic characteristics of the pregnant women. Analysis of age, educational status and marital status, were done for the clients with information recorded in questionnaire.

**Table 1:** Socio-demographic characteristics among antenatal attendees

Characteristics	N (%)
**Age groups**	
<21 years	1310 (28.5)
21–25 years	1598 (34.8)
26–30 years	990 (21.5)
31–35 years	445 (10.8)
>35 years	202 (4.4)
**Educational status**	
Primary	3157 (69.4)
Secondary	1219 (26.8)
Tertiary	173 (3.8)
**Marital status**	
Married	4047 (88.9)
Single	491 (10.8)
Widowed	12 (0.3)

The ages for 4,595 pregnant women were recorded and categorized into < 21, 21–25, 26–30, 31–35 and > 35 years. The majority (34.8%) were aged 21–25 years while 4.4% were aged above 35 years. Most (69.4%) of the respondents had primary school education with 3.8% having tertiary education. Of the 4,550 ANC attendees, 81.9% were in a monogamous relationship, 10.8% were single while 0.3% were widowed.

### HIV seroprevalence

A total of 309 (6.7%) ANC attendees were HIV seropositive in all the hospitals under study. Kitale had the highest proportion (68.6%) followed by Kapsabet (18.1%) and 13.3% from South Nandi Hills. The HIV prevalence per hospital was 15.3%, 3.4% and 2% for Kitale, Kapsabet and Nandi hills respectively. Majority of the HIV seropositive antenatal attendees did not know their HIV status prior to visiting the clinic for antenatal care (85.1%), and were not on anti-retroviral therapy (97.1%) (Table 2).

**Table 2:** Characteristics of HIV seropositive pregnant women

Characteristics	Category	Number (N)	Proportion (%)
HIV status known	No	263	85.1
	Yes	46	14.9
Used antiretroviral drugs	No	299	97.1
	Yes	9	2.9

**Figure 1:**
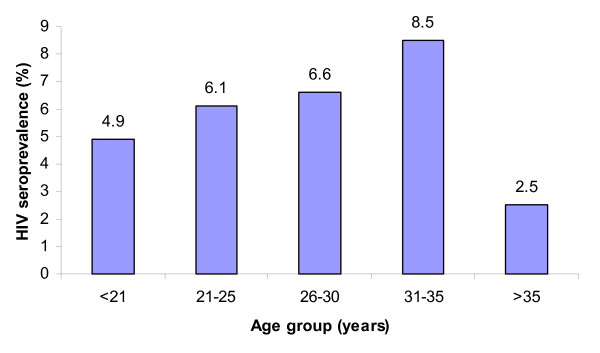
**HIV Prevalence by age group**.

**Figure 2:**
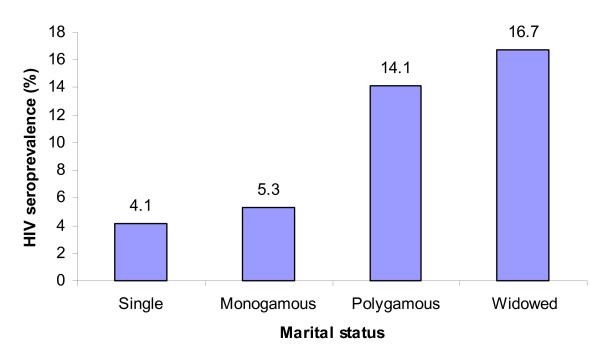
**HIV Prevalence among antenatal attendees by marital status**.

The age specific HIV prevalence among antenatal attendees was analysed and is shown in Figure 1. The 31–35 age group had the highest (8.5%) HIV prevalence, while women aged more than 35 years had the lowest prevalence (2.5%). Widowed antenatal attendees had higher (16.7%) HIV prevalence followed by polygamous, monogamous and single with 14.1%, 5.3% and 4.1% respectively (Figure 2).

**Figure 3:**
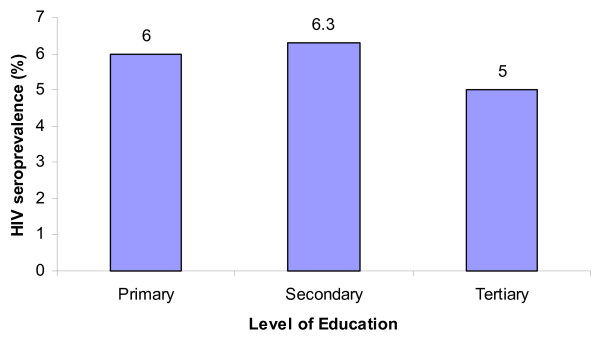
**HIV Prevalence among antenatal attendees by level of education**.

The highest HIV prevalence (6.3%) was recorded among antenatal attendees who had attended secondary schools followed by those with primary and tertiary level of education (6% and 5% respectively) (Figure 3). Table 3 presents the profiles of the pregnant women associated with HIV infection. Women in a polygamous relationship were more likely to be HIV infected as compared to those in a monogamous relationship (p = 0.000). There was no statistically significant association between HIV infection and level of education.

**Table 3:** Profiles of antenatal attendees associated with HIV infection

Characteristics	N (%)	N (HIV Prevalence%)	OR (95% CI)	P-value
**Age groups**				
<21 years	1310 (28.5)	64 (4.89)	1.0	
21–25 years	1598 (34.8)	97 (6.09)	0.8 (0.56–1.1)	0.165
26–30 years	990 (21.5)	65 (6.57)	0.73 (0.51–1.0)	0.083
31–35 years	495 (10.8)	42 (8.49)	0.55 (0.37–0.89)	0.004
>35 years	202 (4.4)	5 (2.48)	2.02 (0.81–5.1)	0.127
**Type of marriage**				
Monogamous	3713 (91.7)	196 (5.28)	1.0	
Polygamous	334 (8.3)	47 (14.07)	0.34 (0.24–0.48)	0.000
**Educational status**				
Primary	3157 (69.4)	188 (5.96)	1.0	
Secondary	1219 (26.8)	77 (6.32)	0.94 (0.71–1.24)	0.653
Tertiary	173 (3.8)	8 (4.62)	1.31 (0.63–2.96)	0.469

### Lymphocyte subset count

The CD4 count was available for 278 ANC attendees. The mean CD4 count was 466 cells/mm^3 ^(9–2000 cells/mm^3^). The CD4 counts were further categorised into three groups (<200, 200–349 and >350 cells per cubic millilitre) based on the WHO CD4/disease staging categories. The majority of the antenatal clinic attendees had >350 cells/mm^3 ^(60.07%) followed by 200–349 cells/mm^3 ^and <200 cells/mm^3 ^with 25.9 and 14.03% respectively (Table 4).

**Table 4:** T-lymphocyte (CD4) count among antenatal attendees

CD4 (cells/mm^3^)	Number (N)	Proportion (%)
<200	39	14.03
200–349	72	25.90
>350	167	60.07
Total	278	100

## Discussion

The HIV prevalence reported in this study is similar to earlier reports in Kitale district hospital and other health facilities used as sentinel surveillance sites for HIV and STDs prevalence in Kenya [2,3]. In the current study, the overall HIV prevalence was 6.7% with Kitale district hospital having the highest (15.3%) followed by Kapsabet and South Nandi Hills district hospitals (3.4% and 2% respectively). In 2001, 2002, 2003 and 2004, the HIV prevalence in Kitale district hospital was 13%, 16%, 11% and 7% respectively among antenatal attendees [3]. The disparities in HIV prevalence in the hospitals may be due to differences in urbanization, ethnic groups and economic activities in the specific areas but further studies are required to elucidate the reasons in details.

The highest proportion of HIV infected women was in the age group 21–25 years (35.5%). This peak age group differed from the report of a demographic health survey carried out in 2003 where the peak was in 25–29 years (13%) among women [2].

In this study, it was found that women in a polygamous marriage had a higher HIV positivity than those in monogamous union (p = 0.000). This suggests that polygamy is a risk factor for HIV-1 infection. However, it should be noted that marital status was self-reported and the study did not specifically request for information on multiple partners. Despite this limitation in our study, it has clearly shown that there is a relationship between HIV status and marital status. This finding is in agreement with previous reports where susceptibility and vulnerability to HIV/AIDS was attributed to marital and family status [4,5].

In the current study, there was no statistical significance between the level of education and HIV infection (p = 0.653 and p = 0.469 for secondary and tertiary education respectively). This differed from previous reports where higher levels of education were associated with a higher HIV seroprevalence [6]. However, some serial cross-sectional studies have found greater reductions in HIV prevalence among the more educated groups, especially in cohorts of young adults [7,8]. These findings suggest that there is a shift in the association between education level and HIV infection.

Among the pregnant women whose CD4 count was determined, 39 (14%) had less than 200 cells per cubic millilitre. As per the current Ministry of Health guidelines on ARV therapy, all these women (39) should be on antiretroviral treatment [9]. However, this study has established that nine of these women are on ARVs, suggesting that there are still major challenges in access of ARVs for those who need them. Such a population would therefore be missing the benefits of timely introduction of HAART, such as the reported threefold reduction of AIDS incidence when HAART is administered to patients with CD4 cell counts below 200 cells/mm^3 ^[10].

It is important to critically evaluate the results and the whole study. The present study has certain limitations that need to be taken into account. The first major limitation is that it was part of a larger study looking into drug resistant HIV genotypes and their effect on prophylaxis against HIV vertical transmission. In this regard, there are other socioeconomic factors such as acres of land owned, number of animals and cultivated farmlands that were not include in the current study. The intricate relationship between poverty and HIV has been shown to be a vicious cycle in response to HIV pandemic. While increasing poverty levels fuel the spread of HIV, the pandemic itself exacerbates those levels in households and families with people living with HIV/AIDS [11]. The limitations of this study bring forth some fruitful and interesting possible avenues for future research that might be needed in relation to the theme of the study.

## Conclusion

Scaling up HIV prevention efforts is mandatory in order to prevent the escalation of the HIV epidemic associated with individuals not knowing their status. The enumeration of lymphocyte subset count should be introduced as a routine test in the antenatal clinic. This will ensure that the HIV seropositive pregnant women can be put on antiretroviral therapy (ART) at an appropriate time.

## Competing interests

The authors declare that they have no competing interests.

## Authors' contributions

All authors contributed to the paper. MK and ES conceived the study. MK conducted the study. EK, ZG, SM and JM supervised the research. All authors reviewed drafts of the manuscript.

## Pre-publication history

The pre-publication history for this paper can be accessed here:

http://www.biomedcentral.com/1472-698X/9/10/prepub

## References

[B1] Barsigo A DeCock KM Chebet KL Marum LH Cheluget B Mwikya L Wandera C Estimating rural seroprevalence using rural sites and women's residence in sentinel surveillance for HIV-1 in Kenya Int Conf AIDS 2002 14

[B2] Central Bureau of Statistics Kenya demographic and health survey 2003 Calverton, Maryland, USA: CBS, MOH, ORC MARCO

[B3] National AIDS and STI Control Programme, Ministry of Health, Kenya AIDS in Kenya: background, projections, impact, interventions and policy 2005 7 Nairobi: NASCOP

[B4] Nyindo M Complementary factors contributing to the spread of HIV-1 in sub-Saharan Africa: a review East African Medical Journal 2005 82 1 40 46 16122111 10.4314/eamj.v82i1.9293

[B5] Dada-Adegboha HO Socio-cultural factors affecting the spread of HIV/AIDS in Africa: a case study Afr J Med Med Sci 2004 33 2 179 182 15565940

[B6] Smith J Nalagoda F Wawer MJ Serwadda D Sewankambo N Konde-Lule J Lutalo T Li C Gray RH Education attainment as a predictor of HIV risk in rural Uganda: results from a population based study International Journal of STD and AIDS 1999 10 7 452 459 10.1258/0956462991914456 10454180

[B7] Michelo C Sandoy IF Fylkesnes K Marked HIV prevalence declines in higher educated young people: evidence from population-based surveys (1995–2003) in Zambia AIDS 2006 20 7 1031 1038 10.1097/01.aids.0000222076.91114.95 16603856

[B8] De Walque D Nakinyingi-Miiro JS Busingye J Whitworth JA Changing association between schooling levels and HIV infection over 11 years in a rural population cohort in south-west Uganda Tropical Medicine and International Health 2005 10 10 993 1001 10.1111/j.1365-3156.2005.01475.x 16185233

[B9] National AIDS and STI Control Programme, Ministry of Health, Kenya Guidelines for Antiretroviral Drug Therapy in Kenya 2005 3 Nairobi: NASCOP

[B10] Bogaards JA Weverling GJ Geskus RB Miedema F Lange JM Bossuyt PM Goudsmith J Low versus high CD4 cell count as starting point for introduction of antiretroviral treatment in resource-poor settings: a scenario-based analysis Antiviral Therapy 2003 8 1 43 50 12713063

[B11] Jefferis K Kinghorn A Siphambe H Thurlow J Macroeconomic and household-level impacts of HIV/AIDS in Botswana AIDS 2008 22 Suppl 1 S113 S119 10.1097/01.aids.0000327631.08093.66 18664942

